# Clonal Hematopoiesis in Patients With Neuroendocrine Tumor Treated With Lutetium-177 and the Risk of Thrombocytopenia: A Prospective Study

**DOI:** 10.1200/PO.24.00143

**Published:** 2024-07-08

**Authors:** Yael Kusne, Terra Lasho, Christy Finke, Zaid Elsabbagh, Shaylene McCue, Timothy Hobday, Jason Starr, Tanios Bekaii-Saab, Thorvardur R. Halfdanarson, Mrinal M. Patnaik, Fang-Shu Ou, Mohamad Bassam Sonbol

**Affiliations:** ^1^ Division of Hematology/Oncology, Mayo Clinic, Phoenix, AZ; ^2^ Division of Hematology, Department of Internal Medicine, Mayo Clinic, Rochester, MN; ^3^ Division of Clinical Trials and Biostatistics, Mayo Clinic, Rochester, MN; ^4^ Division of Medical Oncology, Department of Internal Medicine, Mayo Clinic, Rochester, MN; ^5^ Division of Hematology/Oncology, Mayo Clinic, Jacksonville, FL

## Abstract

**PURPOSE:**

Thrombocytopenia is a relatively common dose-limiting toxicity during peptide receptor radionuclide therapy (PRRT) in patients with NET. Although uncommon, some patients develop persistent cytopenia and eventually therapy-related myeloid neoplasm (t-MN), which has a dismal prognosis. As the indications for PRRT are expanding, it is important to investigate factors that may predict cytopenias during/after PRRT. We prospectively evaluated the prevalence of clonal hematopoiesis (CH) and cytopenia in patients with NET undergoing PRRT.

**MATERIALS AND METHODS:**

Patients with metastatic NET with plan to receive four cycles of lutetium-177 were enrolled. CH was evaluated before PRRT using a panel of 220 genes with a targeted depth of ≥1,000×. Patients were followed during PRRT and every 3 months thereafter.

**RESULTS:**

Of 37 patients enrolled, the median age was 68 years and 51.4% were male. Previous treatment exposures included alkylating agents in 30%, platinum agents in 8%, and external radiation in 13%. CH was detected in 35.1% using a variant allele frequency (VAF) cutoff of ≥2% and 45.9% with a VAF of ≥1%. The most common mutations were in age-related genes (*DNMT3A*, *TET2*). CH was not associated with anemia or neutropenia; however, it was associated with lower platelet count at baseline and more time spent in a thrombocytopenic state during/after PRRT. Five patients had bone marrow biopsies (BMBs) because of sustained hematologic dysfunction post-PRRT, and of those, diagnoses included clonal cytopenia of undetermined significance (CCUS) in three and idiopathic cytopenia of undetermined significance (ICUS) in two.

**CONCLUSION:**

CH is present in 35.1% of patients with NET and is associated with thrombocytopenia risk during PRRT. Future studies with long-term follow-up will delineate whether CH might be a predictor for higher risk of t-MN after PRRT.

## INTRODUCTION

The incidence of neuroendocrine tumors (NETs) has been increasing overtime, and prognosis depends on multiple factors including primary tumor location, disease grade, stage, and rate of differentiation and proliferation.^1-3^ Given that most NETs have high expression of somatostatin receptor (SSTR), somatostatin analogs (SSA) are used as first-line therapy.^4^ Second-line therapies include peptide receptor radionuclide therapy (PRRT), which allows for the delivery of radiopharmaceutical agents directly to tumor cells by targeting SSTRs. In PRRT, the SSA is linked to a specific radioisotope such as Lu-DOTATATE (^177^Lu).^5^ ^177^Lu received FDA approval in 2018 after the NETTER-1 trial showed improved progression-free survival (PFS) with ^177^Lu + SSA compared with SSA alone in patients with progressive gastroenteropancreatic (GEP) NETs.^6,7^ More recently, the NETTER-2 trial showed the efficacy of PRRT in the frontline setting in patients with grade 2 or 3 NET.^8^ Hematotoxicity, and in particular, thrombocytopenia, is a relatively common adverse event of PRRT and is often a limiting factor to receiving a full therapeutic course.^6,9-12^ Furthermore, therapy-related myeloid neoplasms (t-MNs) have been reported post-PRRT with rates ranging from 2% to 20% and a median time from first PRRT to t-MN of 2.8 years.^9,13-19^ Our recent retrospective analysis revealed that among 346 patients treated with at least one cycle of PRRT at Mayo Clinic-Rochester, 4% were diagnosed with t-MN or therapy-related clonal cytopenia of undetermined significance (t-CCUS).^20^ The development of t-MN carries a poor prognosis with a 5-year overall survival (OS) of <10%, and therefore, it is imperative that we elucidate the risks of PRRT.^21,22^ Previous studies have evaluated risk factors for t-MN, including alkylating agents, previous external radiation (RT) exposure, and others, including clonal hematopoiesis (CH).^13,14,19^

**CONTEXT**

**Key Objective**
In patients with neuroendocrine tumors (NETs) who are receiving peptide receptor radionuclide therapy (PRRT), is underlying clonal hematopoiesis (CH) a risk for hematologic toxicity?
**Knowledge Generated**
Prevalence of CH was 35% before PRRT, with age-related CH variants being most common (*DNMT3A, TET2*). Patients with CH had lower baseline platelet counts before PRRT and spent more time thrombocytopenic during PRRT. Post-PRRT, variants in DNA damage response genes were common.
**Relevance**
In patients with NET with CH, PRRT may lead to increased short- and long-term hematologic dysfunction. Screening before PRRT and hematologic monitoring during and after PRRT should be considered.

CH defines somatic mutations of leukemia-associated driver genes within subpopulations of hematopoietic stem cells (HSCs) and is a precursor to myeloid neoplasms. When CH is associated with unexplained cytopenia, it is termed CCUS.^23^ T-CCUS describes unexplained cytopenia with clonal abnormality after DNA-damaging therapy and is associated with progression to t-MN.^24,25^ The progression from CH/CCUS to t-MN depends on mutation, growth rates, context exposures, and the acquisition of additional genetic aberrations.^26-32^ RT exposure has also been associated with t-MN with a median latency of 6.5 years.^33^

While the rate of CH/CCUS has been explored in various solid tumors, the prevalence is not well known in patients with NET.^26,29^ Herein, we hypothesized that PRRT-induced thrombocytopenia may be related to underlying CH in patients with NET. To address this, we prospectively followed 37 patients with NET treated with ^177^Lu to characterize the incidence of cytopenias and CH/CCUS.

## MATERIALS AND METHODS

### Study Population

This prospective study was approved by the Mayo Clinic Institutional Review Board (IRB); informed written consent was provided by patients at enrollment. Inclusion criteria included patients with metastatic NET 18 years and younger undergoing PRRT between September 2020 and May 2022. ^177^Lu was administered intravenously at a dose of 200 mCi once every 8 weeks for four doses.^6^ Complete blood counts were obtained before each treatment and at 3-month follow-up intervals post-PRRT. Toxicity was recorded with the Common Terminology Criteria for Adverse Events (CTCAE), version 5.0. CH and CCUS were defined per 2022 WHO and International Consensus Classification (ICC) criteria.^34,35^ The primary end point of the study was thrombocytopenia (≥grade 1) during treatment and follow-up. Secondary end points included the prevalence of CH and cytopenia at baseline, incidence of t-MN, and overall survival (OS). We chose thrombocytopenia as the primary end point for the following reasons: (1) it is considered the most common hematologic toxicity post-PRRT, (2) nadir usually occurs 4-6 weeks post-PRRT, and (3) almost all patients who develop t-MN had previous thrombocytopenia.^36^

### Next-Generation Sequencing

DNA was extracted from primary mononuclear cells enriched by density gradient centrifugation. Target capture was performed as described.^37-40^ Briefly, libraries were prepared by custom capture using a hybrid-target enrichment covering the entire coding regions for 220 genes. Samples were sequenced using Illumina NovaSeq SP (Illumina, San Diego, CA) to accommodate a targeted depth of ≥1,000×. Initial filters were provided by the Genome Analysis Toolkit (GATK) for single nucleotide and small insertion/deletion variant calling. For clinical NGS performed on bone marrow biopsy (BMB), a 42-gene panel with a sensitivity of 5%-10% and a minimum depth of 250× was used as described.^40^ Variants with minor allele fractions ≥0.6% by GnomAD were excluded. The remaining variants were functionally annotated using ANNOVAR software, and Integrative Genomics Viewer (IGV) was used for manual reviews of alignment for selected variant calls.

### Statistical Analysis

Continuous variables were presented as medians with ranges and compared between CH and no CH patients using the Wilcoxon rank-sum test. Categorical variables were expressed as counts with percentages and compared using the chi-square test. OS was estimated using Kaplan-Meier and compared using the log-rank test. A multistate model was used to investigate longitudinal changes in blood counts.^41^ Illness-death models were used where patients can transition between healthy and illness (eg, thrombocytopenic), with death as the absorbing state. Probability in state and time spent in the state (ie, sojourn time) were estimated using Aalen-Johansen estimator.^42^ Multistate hazards model was used to estimate the transition rate while adjusting for bone mets. Additional details are given in the Data Supplement. Analyses were performed using SAS (version 9.4; SAS Institute, Cary, NC) and R (version 4.2.2). Two-sided *P* values of <.05 were considered statistically significant, and there was no adjustment for multiple comparisons.

## RESULTS

### Patient Characteristics

A total of 37 patients (51.4% male) with a median age of 68 years (range, 34-85) were enrolled (Table 1). Most patients had small-bowel NET (n = 19, 51.4%), all patients had stage IV well-differentiated NET, and 81.1% (n = 30) had grade 1 or 2 disease. About one third (n = 11, 29.7%) had previous exposure to temozolomide or platinum agents, and 13.5% (n = 5) had a history of RT (4 to bone mets, one for lymphoma 40 years ago to a nonbony site). Among patients with a history of chemotherapy (chemo) or RT, the median time from first chemo to NGS was 2.5 years (range, 0.23-14.7) and the median time from first RT to NGS was 4.8 years (range, 0.3-40.5).

**TABLE 1.** Patient Characteristics

Clinical Value	No CH (n = 24)	CH (≥2%) (n = 13)	Total (n = 37)	*P*
Age, years, median (range)	68.0 (34.0-81.0)	69.0 (59.0-85.0)	68.0 (34.0-85.0)	.60^a^
Male, No. (%)	14 (58.3)	5 (38.5)	19 (51.4)	.25^b^
Primary tumor type, No. (%)				.71^b^
Gastric	1 (4.2)	0 (0.0)	1 (2.7)	
Head/neck	0 (0.0)	1 (7.7)	1 (2.7)	
Other	2 (8.3)	1 (7.7)	3 (8.1)	
Pancreatic	8 (33.3)	3 (23.1)	11 (29.7)	
Paraganglioma	1 (4.2)	1 (7.7)	2 (5.4)	
Small bowel	12 (50.0)	7 (53.8)	19 (51.4)	
Grade, No. (%)				.46^b^
1	5 (20.8)	3 (23.1)	8 (21.6)	
2	15 (62.5)	7 (53.8)	22 (59.5)	
3	2 (8.3)	0 (0.0)	2 (5.4)	
Unknown	2 (8.3)	3 (23.1)	5 (13.5)	
Ki-67%, median (range)	7.3 (1.0-65)	8.5 (2.0-20)	8.5 (1.0-65)	.54^a^
Bone mets, No. (%)	9 (37.5)	10 (76.9)	19 (51.4)	.02^b^
Severity of bone mets, No. (%)				.07^b^
None	15 (62.5)	3 (23.1)	18 (48.6)	
Mild	5 (20.8)	6 (46.2)	11 (29.7)	
Severe	4 (16.7)	4 (30.8)	8 (21.6)	
Previous therapy	6 (25.0)	5 (38.5)	11 (29.7)	.39^b^
Alkylating	2 (8.3)	1 (7.7)	3 (8.1)	.95^b^
Platinum mTOR inhibitor	4 (16.7)	4 (30.8)	8 (21.6)	.32^b^
Somatostatin analog	22 (91.7)	13 (100)	35 (94.6)	.28^b^
External radiation	2 (8.3)	3 (23.1)	5 (13.5)	.21^b^
Baseline laboratory tests				
Hemoglobin (g/dL), median (range)	13.0 (10.6-16.3)	13.3 (10.7-15.4)	13.0 (10.6-16.3)	.57^a^
RDW (%), median (range)	13.9 (11.9-21.3)	14.1 (12.4-17.6)	14.0 (11.9-21.3)	.76^a^
MCV (fL), median (range)	91.0 (76.5-100.2)	92.5 (88.7-96.0)	91.4 (76.5-100.2)	.35^a^
Platelets (×10^9^/L), median (range)	230.5 (153-473)	184.5 (113-367)	220.0 (113-473)	.03^a^
Leukocytes (×10^9^/L), median (range)	6.4 (4.0-12.7)	6.0 (2.4-8.8)	6.1 (2.4-12.7)	.43^a^
Neutrophils (×10^9^/L), median (range)	4.4 (2.0-10.0)	3.7 (1.3-7.3)	3.8 (1.3-10)	.51^a^
Lymphocytes (×10^9^/L), median (range)	1.3 (0.4-2.3)	1.5 (0.5-2.0)	1.4 (0.4-2.3)	.46^a^
Completed four cycles, No. (%)	17 (70.8)	8 (61.5)	25 (67.6)	.56^b^
Total GBq received, median (range)	789.1 (187-840)	765.2 (211-841)	781.2 (187-841)	.17^a^
12-month laboratory tests				
Hemoglobin (g/dL), median (range)	12.1 (7.4-17.0)	12.6 (9.4-17.2)	12.5 (7.4-17.2)	.88^a^
RDW (%), median (range)	14.1 (12.0-21.0)	13.2 (12.2-16.5)	14.1 (12.0-21.0)	.29^a^
MCV (fL), median (range)	97.1 (64.7-111.0)	97.1 (91.1-101.0)	97.1 (64.7-111.0)	.77^a^
Platelets (×10^9^/L), median (range)	169.0 (36.0-264.0)	199.5 (103.0-427.0)	172.0 (36.0-427.0)	.43^a^
Leukocytes (×10^9^/L), median (range)	4.4 (1.8-13.0)	5.5 (3.6-9.2)	5.4 (1.8-13.0)	.72^a^
Neutrophils (×/L), median (range)	2.8 (1.1-5.1)	4.0 (2.4-4.7)	2.8 (1.1-5.1)	.16^a^
Lymphocytes (×10^9^/L), median (range)	0.7 (0.3-2.2)	0.8 (0.3-2.7)	0.8 (0.3-2.7)	.60^a^

Abbreviations: CH, clonal hematopoiesis; MCV, mean corpuscular volume; mTOR, mammalian target of rapamycin; RDW, red cell distribution width.

^a^ Wilcoxon rank-sum *P* value.

^b^ Chi-square *P* value.

### Prevalence of CH Before PRRT

**FIG 1.**
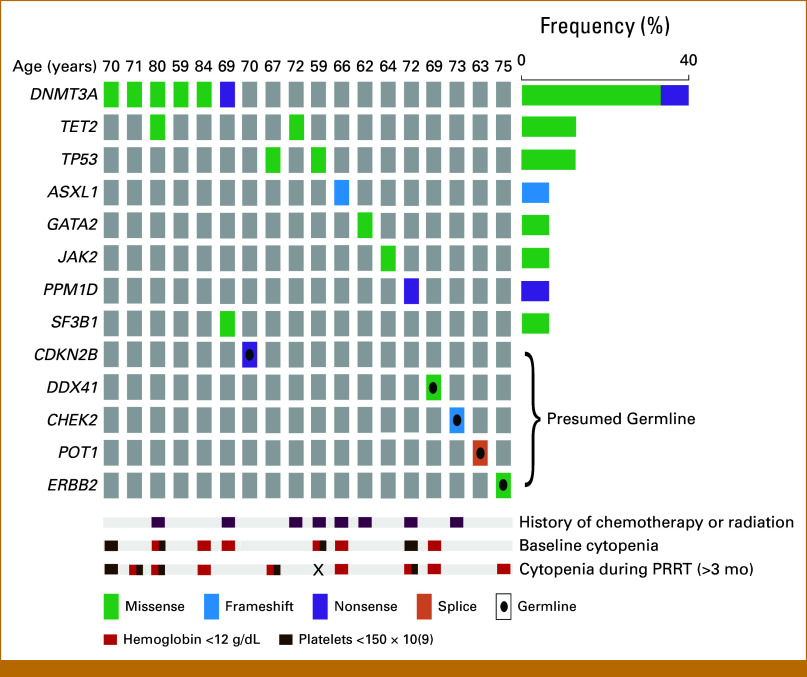
Oncoplot of pathogenic variants, previous therapies, and cytopenias among patients with variants detected with VAF ≥2%. The overall prevalence of CH was 35%. One patient did not receive PRRT, and therefore, cytopenia could not be evaluated during treatment (indicated by X). Variants with VAF ≥2% were considered CH. PRRT, peptide receptor radionuclide therapy.

Of the 37 patients, 28 pathogenic variants were identified among 21 patients (Fig 1). Five (18%) of the 28 variants were presumed germline on the basis of VAF (*CDKN2B, DDX41, CHEK2, POT1, ERBB2*; median VAF 46.5%, range, 44.0%-52.1%; Fig 1).^43,44^ Eight (28.5%) of the 28 pathogenic variants had a VAF of <2%. In line with the commonly accepted threshold of VAF ≥2%,^34^ and after excluding the presumed germline variants (n = 5), the prevalence of CH was 35.1% (15 variants among 13 patients, of 37 sequenced). When evaluating patients with VAF ≥1%, the prevalence was 45.9%. Among those with VAF ≥2% (n = 13), the median VAF of all pathogenic variants was 7.3% (range, 2.0%-50.30%). The most common pathogenic variant with VAF ≥2% was in *DNMT3A* (n = 6), followed by *TET2* (n = 2). Patients with bone mets were more likely to have CH (*P* = .02) although severity of bone metastatic burden was not associated with CH (*P* = .07, Table 1). Previous history of chemo or RT was not associated with CH (Table 1).

### Baseline Cytopenias

**FIG 2.**
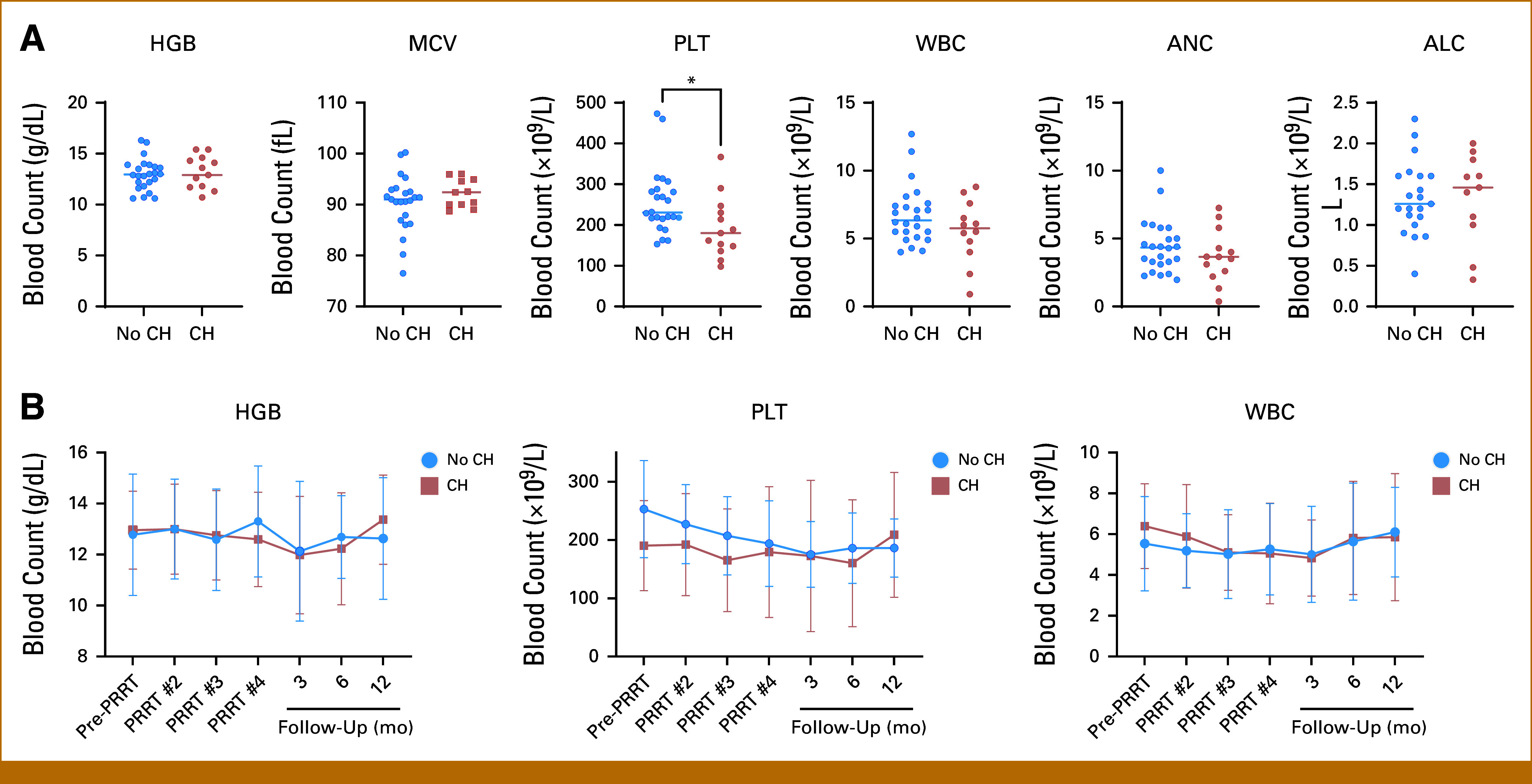
Patients with NET with CH had a lower platelet count at baseline. (A) Baseline blood counts. Patients with CH (VAF ≥2%) had lower platelet counts than those without CH (*P* = .03). There were no differences in HGB, MCV, WBC, ANC, or ALC. The horizontal line depicts median. (B) During four cycles of PRRT and at 3-month, 6-month and 12-month follow-ups, there was no significant difference in blood counts between groups. Mean with SD at each cycle and follow-up are shown. ALC, absolute lymphocyte count; ANC, absolute neutrophil count; CH, clonal hematopoiesis (defined by VAF ≥2%); Hgb, Hemoglobin; MCV, mean corpuscular volume; NET, neuroendocrine tumor; PLT, platelet; SD, standard deviation.

We first investigated whether patients with CH were more likely to have cytopenias at baseline pre-PRRT. There were no statistically significant differences in baseline hemoglobin (*P* = .57), red cell distribution width (RDW, *P* = .76), mean corpuscular volume (MCV, *P* = .35), leukocytes (*P* = .43), neutrophils (*P* = .51), or lymphocytes (*P* = .46) between those with CH and those without (Table 1, Fig 2A). However, patients with CH (VAF ≥2%) had a lower platelet count at baseline with a median of 185 × 10^9^/L (range, 113-367) compared with 231 × 10^9^/L (range, 153-473) in those without CH (*P* = .03, Fig 2A).

### PRRT and Toxicity

**FIG 3.**
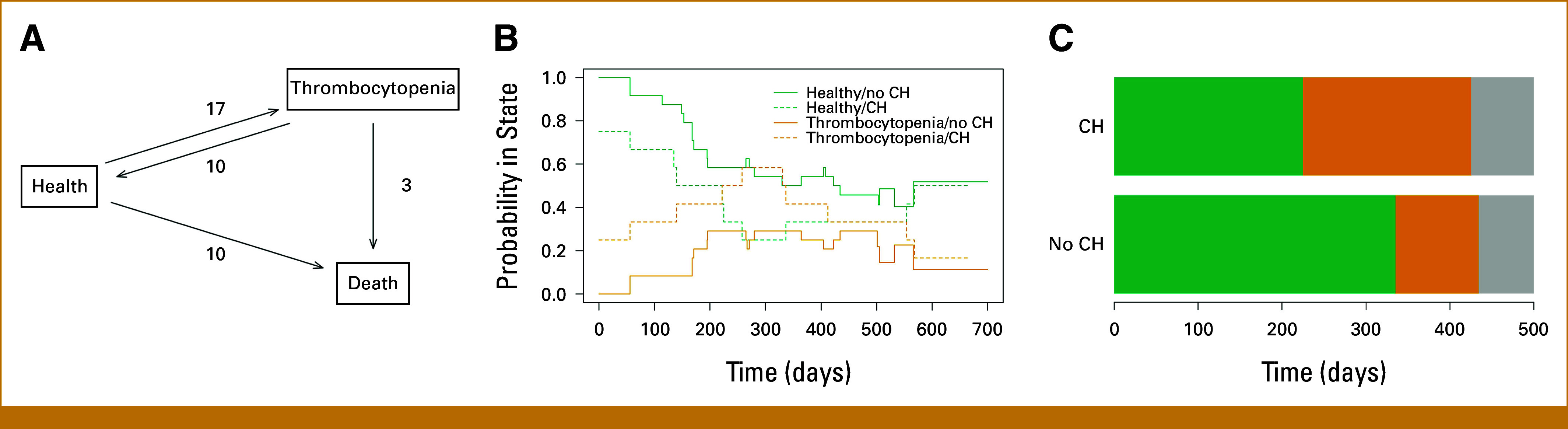
Patients with NET with CH were more likely to be thrombocytopenic during PRRT. (A) State space and transition counts for thrombocytopenia depict patient transitions from normal platelet count (health) to any grade of thrombocytopenia or death. (B) Aalen-Johansen plot showing that patients with CH (VAF ≥2%) were more likely to experience thrombocytopenia and remain thrombocytopenic longer than patients without CH. (C) On average, patients with CH spent about 200 days (95% CI, 88.3 to 312.4) being thrombocytopenic compared with 99 days (95% CI, 45.3 to 153.3) without. Green represents healthy, orange represents thrombocytopenia, and gray represents death. CH, clonal hematopoiesis.

The median follow-up was 26.0 months (IQR, 21.2-29.0). Most patients received four PRRT treatments (n = 25, 67.6%), with a total median dose of 781 mCi (range, 187-841). One patient (ID #4) did not receive any PRRT because of cytopenias, comorbidities, and prolonged hospitalization, which ultimately resulted in death. Overall, two patients discontinued PRRT after three cycles because of cytopenias and three patients required transfusions during PRRT treatment (all without CH). During PRRT and follow-up, there were no statistically significant differences in cytopenias between groups (Fig 2B), including in grade 3 or 4 cytopenias. Thirteen patients transitioned from normal platelet count (health) to thrombocytopenia once, and two patients made the same transition twice (Fig 3A). Notably, there were 10 instances of patients who recovered from thrombocytopenia to health, whereas 10 patients died without experiencing thrombocytopenia. Patients with CH had a higher probability of being thrombocytopenic than patients without CH overtime (Fig 3B). The highest probability of thrombocytopenia, among patients with CH, was 58.3%, which was observed about 300 days after starting PRRT (Fig 3B). CH was not associated with an increased incidence of thrombocytopenia (HR, 0.73 [95% CI, 0.26 to 2.01]), but patients with CH were less likely to recover from thrombocytopenia to normal platelets (HR, 0.17 [95% CI, 0.04 to 0.66]) compared with patients without CH. On average, patients with CH spent more time thrombocytopenic (200 days, [95% CI, 88 to 312]) than patients without CH (99 days [95% CI, 45 to 153]), restricting to 500 days after initiation of PRRT, and this was true for grade ≥3 thrombocytopenia as well (8 days more [95% CI, –16.2 to 32.1]; Figs 3C, Data Supplement, Fig S1). There were no differences across groups for anemia, leukopenia, neutropenia, or lymphopenia (Data Supplement, Fig S2). The 1-year OS rate was 75.0% and 79.2% for those with and without CH, respectively (*P* = .77), whereas median was not reached (Data Supplement, Fig S3).

### CH Assessment Post-PRRT

In total, five patients (13.5%) enrolled had BMB performed post-PRRT because of persistent cytopenias with a median time from first PRRT to BMB of 1.13 years (range, 0.8-1.3) (Table 2, Data Supplement, Fig S4). Of these 5, one patient (ID #18) had a history of temozolomide and RT to bone mets (femur, hip, and thoracic spine). Pre-PRRT, ID #18 had one *TET2* pathogenic variant detected with a VAF of 1.5%, which was not detected on post-PRRT NGS, likely because of differences in sequencing sensitivities. ID #2, who had a history of lymphoma treated with single-agent RT 40 years before PRRT, had a low-level pathogenic *PPM1D* variant (c.1433dupG) with a VAF of 0.80% detected pre-PRRT; and on post-PRRT BMB, the same variant was detected with a VAF of 18.9%.

**TABLE 2.** Characteristics of Patients With NET With Post-PRRT CH Data Available

ID	Previous Chemotherapy or RT	Pre-PRRT NGS (blood)	PRRT Cycles	Cytopenia During/After PRRT	Time Between Enrollment and BMB	Karyotype	NGS (BM)^a^	Diagnosis	Outcome
#3	No	*DNMT3A* c.2014del (1.5%) *DNMT3A* c.1906G>T (3.3%)	4	Gr1 thrombocytopenia and anemia >6 mo	13.6 mo	46,XY[20]	None detected	ICUS *v* CCUS^b^	Stable
#9	No	*DNMT3A* c.2396C>A (3.9%)	3	Gr2 anemia Gr3 thrombocytopenia	10.8 mo	46,XY[12]	*PPM1D* c.1434C>A; p.C478* (3%) *PPM1D* c.1627dup; p.S543Ffs*9 (2%) *DNMT3A* c.2396C>A; p.P799H (26%)	t-CCUS	Deceased
#2	RT 40 years before for lymphoma (nonbony site)	None detected	3	Gr2 anemia >6 mo	10.0 mo	46,XY[20]	*PPM1D* c.1433dup; p.C478fs* (19%)	t-CCUS	Deceased
#18	TMZ and RT to bone mets	*TET2* c.1454_1457dupTGAA (1.3%)	2	None	13.6 mo	46,XX[20]	None detected	ICUS *v* CCUS^b^	Stable
#30	No	None detected	4	Gr1 anemia Gr2 thrombocytopenia	16.0 mo	46,XY[20]	*PPM1D* c.1608delG; T537Hfs*2 (14%) *PPM1D* c.1654C>T; R552* (11.7%)	t-CCUS	Stable

Abbreviations: BMB, bone marrow biopsy; ICUS, idiopathic cytopenia of undetermined significance; ID, patient identifier; NGS, next generation sequencing; RT, radiotherapy; t-CCUS, therapy related clonal cytopenia of undetermined significance; TMZ, temozolomide.

^a^ NGS assay used includes 42 genes; sensitivity is reported as 5%-10% VAF with a minimum depth of 250×.

^b^ ID #3 and #18 were classified as ICUS, as on the basis of the time of BMB, no variants were detected. Notably, ID #3 had two *DNMT3A* variants detected with VAFs of 1.5% and 3.3% and ID #18 had one *TET2* variant detected with a VAF of 1.5%. This discrepancy is likely due to variations in sequencing sensitivity.

BMB evaluations were consistent with diagnoses of idiopathic cytopenia of undetermined significance (ICUS) in two patients (ID #3 and #18) as no variants were detected on NGS at the time of BMB. Importantly, both these patients had low-level variants detected pre-PRRT. ID #3 had two pathogenic *DNMT3A* variants (c.1906G>T, c.2014del) detected pre-PRRT with VAFs of 3.3% and 1.5%; however, post-PRRT, these variants were not detected. ID #18 had one pathogenic variant in *TET2* (c.1454_1457dupTGAA) detected pre-PRRT, which was not detected on post-PRRT BMB sequencing. In both cases (ID #3, #18), it is likely that these variants were not detected because of differences in sequencing sensitivities. Three other patients (ID #9, #2, #30) were diagnosed with CCUS (Table 2, Data Supplement, Fig S4), and of those three, NGS revealed five *PPM1D* pathogenic variants, one of which was detected pre-PRRT (ID #2, c.1433dupG) at a low VAF of 0.80%, whereas the other four variants were not detected pre-PRRT. ID #9 had a *DNMT3A* variant pre-PRRT (c.2396C>T) with a VAF of 3.9%, and this variant was detected on post-PRRT NGS with a VAF of 26%.

## DISCUSSION

In patients with NET treated with ^177^Lu, reports of t-MN have indicated variable incidence, with rates as high as 20%.^9,13-15,19,20,45-47^ Previous studies have assessed potential risk factors for t-MN after PRRT; however, there have been no significant associations across different studies.^13,14,19^ Here, we evaluated CH in patients with NET before receiving ^177^Lu-DOTATATE and prospectively followed them over time. CH was present in 35% of patients when a VAF cutoff of 2% was used and 46% with a cutoff of 1%. This is consistent with a French study where 46.5% (27 of 58) of patients with NET were found to have CH.^45^ In addition, a smaller study of 13 patients with NET recently reported a CH prevalence of 62% (8 of 13) with a VAF cutoff of ≥1%.^48^ This is in comparison with other solid tumors where the prevalence of CH has been reported at 30%.^26^ It is important to note, however, that in addition to VAF cutoff, the prevalence of these mutations depends on age and method of sequencing, as more sensitive methods can detect mutations at lower frequencies.^49^

While somatic mutations in the epigenetic modifier genes *DNMT3A, TET2, ASXL1* (termed DTA) are most common in the general aging population, patients with a history of cytotoxic chemo and/or RT exposure have an increased incidence of CH in DNA damage response (DDR) genes (*PPM1D, ATM2, TP53, CHEK2*).^26,29,50^ Pathogenic mutations in DDR genes are significantly more common in t-MN when compared with primary de novo myeloid neoplasms,^50^ and previous reports in patients with NET have shown that PRRT exposure led to clonal expansion of these DDR genes.^26,45,48^ In our study, DTA mutations were most common, in line with general population data. Interestingly, among the patients included in our cohort with post-PRRT CH data available, 60% (3 of 5) developed new *PPM1D* mutations on bone marrow NGS, consistent with the abovementioned reports of clonal expansion of DDR genes post-PRRT. It remains to be seen whether DDR gene variants are present before PRRT, perhaps at low levels, and are selected for by PRRT leading to clonal expansion during PRRT, or whether PRRTs are inducing new DNA damage leading to these variants. While our data would suggest that these *PPM1D* mutations are new, given that they were not present in the three patients who seemed to acquire them post-PRRT, differences in sequencing technique and sensitivity are likely contributory. With a median follow-up of 26.0 months, no patients have been diagnosed with t-MN thus far in our study although of the five patients, 3 (60%) were diagnosed with t-CCUS. Prospective evaluation is ongoing in these patients.

Our study highlights the prevalence of CH in patients with NET. Patients with CH do not have a higher risk of developing thrombocytopenia compared with those without CH; however, once thrombocytopenic, patients with CH are less likely to recover and therefore spend more time in a thrombocytopenic state. Such patients are potentially at risk of developing t-MN when they acquire further genetic mutations.^26^ However, it is important to note that these patients receive multiple chemotherapeutic agents and RT, which have been associated with CH.^26^ Our data, and the work of others, support the hypothesis of CH as an important risk factor for t-MN in patients with NET planned for PRRT. Further follow-up with longitudinal CH assessment post-PRRT is important to fully elucidate this relationship between CH and t-MN post-PRRT.

Identifying patients at risk of t-MN before PRRT is essential as the prognosis of NET patients is excellent and estimated to be in years. The 5-year follow-up data from the original NETTER-1 trial revealed that 2% of the patients developed refractory cytopenias leading to the diagnosis of t-MN. Importantly, the NETTER-1 study included patients with no previous exposure to other systemic therapies except somatostatin analogs. In addition, primary analysis of the NETTER-2 study has already reported one patient with t-MN (0.7%) compared with none in the SSA arm.^8^ Recent studies combining PRRT with temozolomide reported ≥ grade 3 hematologic toxicity in up to 31% of patients, and a phase III study is ongoing using this regimen.^51-53^ Retrospective studies of patients who received both PRRT and capecitabine with temozolomide revealed that 8% developed MDS/AML with a median time to event of 2.8 years.^19,54^ In addition to combination therapy, others have even recommended the use of poly(ADP-ribose) polymerase (PARP) inhibitors to act as a potentiator of PRRT.^55^ However, PARP inhibitors have been linked to CH and t-MN^56^ with a mechanism potentially related to clonal expansion of DDR CH.^26^ Consequently, the combination of PRRT and PARP inhibitors poses a significant potential risk of t-MN in these patients.

The indications for PRRT are expanding. Recently, the VISION trial revealed the clinical efficacy of PRRT in patients with prostate cancer, leading to its approval.^57^ To our knowledge, there are no reports of t-MN after PRRT for prostate cancer; however, this may be due to the relatively recent approved indication. The high rates of hematologic toxicity are concerning that long-term follow-up may reveal increased incidence of t-MN especially in the relatively older population with prostate cancer where PARP inhibitors may be indicated.

Given that reports have shown patients who develop t-MN post-PRRT have a dismal prognosis with a reported median OS of only 13 months,^58,59^ it is imperative that patients who are at risk of t-MN are identified early and counseled on this risk. In this study, we obtained NGS using a comprehensive gene panel with high sensitivity in an unselected group of patients with NET (to our knowledge, the largest prospective sample) with no known previous hematologic malignancies and prospectively followed them during PRRT. Our results indicate that CH may indeed be a risk for hematologic dysfunction during PRRT. However, further studies are needed with larger sample sizes to fully characterize these variants and their impact on hematologic toxicities. Identifying patients who are more likely to experience cytopenias before PRRT may allow for proactive management to avoid treatment delays. In addition, whether bone mets increase the hematologic toxicity risk of PRRT should be explored further. Novel molecular predictors of PRRT activity such as the PRRT predictive quotient (PPQ) have the potential to select the patients most likely to derive benefit from PRRT, and in combinations with predictors of risk, we may ultimately be able to select patients who are likely to benefit from PRRT and have a low risk of severe hematologic complications such as t-MN.^60,61^

One limitation of our study lies in the relatively short follow-up duration, which may constrain the comprehensive assessment of longer-term outcomes such as t-MN. The median follow-up of 26.0 months provides valuable insights into the immediate effects of PRRT on CH and cytopenias. As such, our study's shorter follow-up duration limits our ability to capture the full spectrum of potential hematologic consequences, particularly those with prolonged latency periods. In addition, the prevalence of CH largely depends on the sequencing method, and therefore, it is possible that low-level variants exist and were not detected in our study. The small sample size limits our analysis to mostly descriptive, and all conclusions should be interpreted as exploratory and discovery in nature. Large-scale prospective studies with longer follow-up duration are warranted.

In conclusion, we have shown that among patients with NET, the prevalence of CH (VAF ≥2%) is 35%, with DTA gene variants being most common. Patients with CH had lower platelet counts at baseline compared with patients without CH and remained thrombocytopenic longer on average. In addition, we identified DDR genes in 3 of 5 patients with post-PRRT samples available. With rates of t-MN post-PRRT being reported as high as 20%, long-term follow-up is essential.
